# Quantitative high-throughput screening identifies small-molecule modulators of Wnt/β-catenin signaling

**DOI:** 10.3389/fphar.2026.1788041

**Published:** 2026-04-02

**Authors:** Xing Chen, Shu Yang, Srilatha Sakamuru, Chainarong Sukhawanit, Savannah Wood, Masato Ooka, Ruili Huang, Menghang Xia

**Affiliations:** Division of Preclinical Innovation, National Center for Advancing Translational Sciences (NCATS), National Institutes of Health (NIH), Rockville, MD, United States

**Keywords:** brefeldin A, high-throughput screening, inhibitor, Wnt signaling, β-catenin

## Abstract

The Wnt/β-catenin signaling pathway plays an important role in development and tissue homeostasis, and its dysregulation is implicated in various pathologies, including cancer, fibrosis, and neurodegeneration. However, the discovery of small-molecule modulators of this pathway remains challenging due to the pathway’s inherent complexity, characterized by ligand redundancy, overlapping receptor usage, and compensatory downstream signaling. In this study, we optimized a cell-based LEF/TCF-β-lactamase reporter assay for quantitative high-throughput screening in a 1536-well format. Screening 1280 compounds from the Library of Pharmacologically Active Compounds alongside 88 compounds from the Tox21 collection identified twelve potential antagonists of Wnt/β-catenin signaling. Follow-up studies confirmed the activity of 10 compounds, demonstrating consistent activity across two independent reporter systems (β-lactamase and luciferase). Western blot analysis showed that all compounds except for cytosine-1-beta-D-arabinofuranoside and PMEG reduced accumulation of both non-phosphorylated β-catenin (active) and total β-catenin, providing orthogonal validation of pathway inhibition. The identification of known Wnt inhibitors such as emetine, tyrphostin A9, niclosamide, ouabain, and podophyllotoxin further validated the assay’s robustness. Collectively, this study establishes a robust 1536-well screening platform for identifying Wnt pathway modulators and identifies topotecan, amsacrine, brefeldin A, and tyrphostin AG 879 as candidate small-molecule antagonist, thereby expanding the chemical tools for investigating Wnt/β-catenin signaling.

## 1 Introduction

The Wnt signaling network is broadly divided into canonical (β-catenin-dependent) and non-canonical pathways. Noncanonical pathways, including Wnt/Ca^2+^ and planar cell polarity signaling, function independently of lymphoid enhancer-binding factor/β-catenin-T-cell factor (LEF/TCF) transcriptional activity (Liu et al., 2022). In contrast, the canonical Wnt/β-catenin pathway is characterized by the stabilization and nuclear translocation of β-catenin, which subsequently forms complexes with LEF/TCF transcription factors to activate target gene expression (Liu et al., 2022). This pathway is essential for embryonic development, tissue regeneration and adult homeostasis (Klaus and Birchmeier, 2008), and its dysregulation is a significant contributor to numerous human diseases.

**FIGURE 1:**
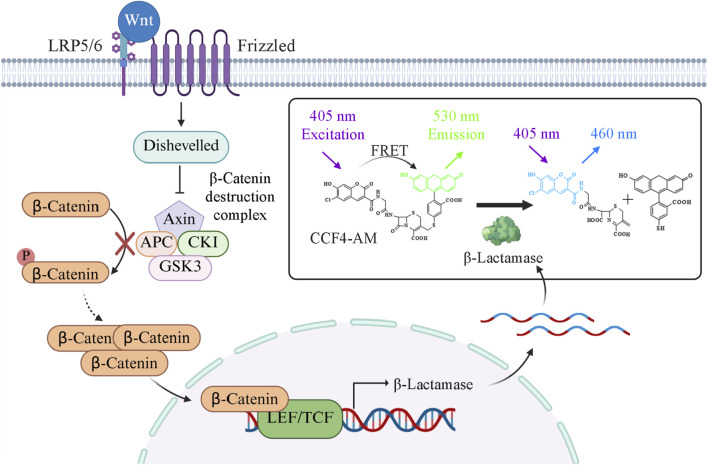
Wnt signaling pathway and schematic of the LEF/TCF reporter gene assay principle. The canonical Wnt signaling pathway begins when Wnt ligands bind to Frizzled receptors and low-density lipoprotein receptor-related proteins 5/6 (LRP5/6) on the cell surface. This interaction inhibits the β-catenin destruction complex, preventing β-catenin phosphorylation, ubiquitination, and proteasomal degradation. Stabilized β-catenin accumulates in the cytoplasm and translocates into the nucleus, where it interacts with lymphoid enhancer-binding factor/T-cell factor (LEF/TCF) transcription factors to activate Wnt target gene expression. In the LEF/TCF-β-lactamase reporter assay, activation of the Wnt/β-catenin pathway leads to transcription of the β-lactamase reporter gene under control of LEF/TCF response elements. The expressed β-lactamase enzyme cleaves the CCF4-AM substrate, producing a measurable fluorescence emission shift (460/530 nm) that reflects pathway activation or inhibition.

Canonical Wnt signaling is initiated when WNT ligands bind to Frizzled receptors and low-density lipoprotein receptor-related protein 5/6 (LRP5/6) co-receptors (Hu et al., 2024). Ligand binding triggers signal transduction via Dishevelled, leading to the inhibition of a cytoplasmic destruction complex composed of Axin, adenomatous polyposis coli (APC), glycogen synthase kinase 3 (GSK3), and casein kinase 1 (CK1) (Liu et al., 2022; Log et al., 2004). Disruption of this complex prevents β-catenin phosphorylation, ubiquitination, and subsequent proteasomal degradation. As a result, β-catenin becomes stabilized and accumulates in the cytoplasm. Accumulated β-catenin then translocate to the nucleus, where it interacts with LEF/TCF factors to activate gene transcription (Log et al., 2004; MacDonald et al., 2009) (Figure 1).

During embryogenesis, Wnt signaling orchestrates critical cellular processes such as cell fate specification, proliferation, migration, and tissue polarity (Cleve et al., 2012). In adults, it is critical for maintaining stem cell populations, facilitating tissue repair, and preserving overall homeostasis (Clevers et al., 2014; Xue et al., 2025). Aberrant Wnt signaling is strongly linked to a range of diseases, such as cancer, fibrosis, osteoporosis, metabolic disorders, and neurodegeneration (Liu et al., 2022; Hu et al., 2024; De Ferrari and Moon, 2006). Notably, mutations in core components like APC or β-catenin are frequently observed in colorectal cancer and other malignancies, driving constitutive activation and contributing to tumorigenesis (Groenewald et al., 2023; Zhan et al., 2017; Anastas and Moon, 2013).

Given its central role in disease, the Wnt pathway is a major therapeutic target (Anastas and Moon, 2013; Kahn, 2014). Antagonists that suppress hyperactive Wnt signaling are actively pursued for oncology indications (Menon et al., 2025; Martin-Orozco et al., 2019; Wang et al., 2024), while agonists have potential in regenerative medicine (Bonnet et al., 2021; Canalis, 2013). However, drug discovery efforts have been hampered by the pathway’s functional redundancy, complexity, and essential physiological roles, leading to off-target toxicities and the emergence of resistance (Kahn, 2014; Park and Kim, 2023; Nusse and Clevers, 2017; Zhong and Virshup, 2020; Hushmandi et al., 2025).

High-throughput screening (HTS) is a powerful approach for identifying small-molecule modulators of signaling pathways (Macarron et al., 2011; Inglese et al., 2006). The adoption of the 1536-well format for quantitative HTS (qHTS) offers increased throughput, reduced reagent consumption, and lower costs (Macarron et al., 2011; Inglese et al., 2006). Nevertheless, successful miniaturization requires careful optimization to ensure adequate sensitivity, dynamic range, and statistical robustness, as measured by parameters, such as signal-to-background (S/B) ratio, coefficient of variation (CV), and Z-factor (Zhang et al., 1999).

Herein, we report the comprehensive development, optimization, and validation of a β-lactamase reporter assay for the Wnt/β-catenin pathway, miniaturized to a 1536-well format suitable for qHTS. Using a HEK293 cell line stably expressing a β-lactamase reporter gene under the control of LEF/TCF response elements, we employed this optimized platform to screen a LOPAC library. This approach enables the efficient identification of potential small-molecule modulators of Wnt/β-catenin signaling for further mechanistic characterization (Figure 1).

## 2 Materials and methods

### 2.1 Reagents and compounds

Recombinant human WNT-3A (hWNT-3A) and R-spondin 2 (hRS) were obtained from R&D Systems (Minneapolis, MN, USA). Lithium chloride (LiCl) was purchased from Sigma-Aldrich (St. Louis, MO, USA). Recombinant human vitronectin (hVTN-N; Cat #A14700), Opti-MEM medium (Cat #11058-021), Dulbecco’s Modified Eagle Medium (DMEM; Cat #10569-010), dialyzed fetal bovine serum (FBS; Cat #26400-044), non-essential amino acids (NEAA; Cat #11140-050), HEPES buffer (Cat #15630-080), penicillin-streptomycin (Pen/Strep; Cat #15140-122), sodium pyruvate (Cat #11360-070), blasticidin (Cat #A11139-03), β-lactamase detection reagents, including CCF4-AM substrate (Cat #K1028) and the β-lactamase Loading Solutions kit (Cat #K1026), were purchased from Thermo Fisher Scientific (Carlsbad, CA, USA). All test compounds were dissolved in dimethyl sulfoxide (DMSO) to prepare stock solutions.

### 2.2 Cell line and culture conditions

The CellSensor® LEF/TCF-bla FreeStyle™ 293F cell line (Invitrogen), which stably expresses a β-lactamase reporter gene under control of a LEF/TCF response element, was used to monitor canonical Wnt/β-catenin signaling activation. Cells were maintained in DMEM supplemented with 10% dialyzed FBS, 0.1 mM NEAA, 25 mM HEPES, 1% Pen/Strep, and 5 μg/mL blasticidin at 37 °C in a humidified atmosphere with 5% CO_2_.

### 2.3 LEF/TCF-β-lactamase (LEF/TCF-bla) reporter assay

LEF/TCF-bla cells were harvested and resuspended in assay medium consisting of Opti-MEM supplemented with 0.5% dialyzed FBS, 0.1 mM NEAA, 1 mM sodium pyruvate, 10 mM HEPES, and 1% Pen/Strep. To enhance cell adherence within the 1536-well plates (Dai et al., 2016), hVTN-N was added to cell assay medium at a 1:300 ratio prior to dispensing. A Multidrop Combi Reagent Dispenser (Thermo Fisher Scientific) was used to dispense cells into 1536-well black, clear-bottom tissue culture plates (Greiner Bio-One) at a density of 2,500 cells in 5 µL per well. Assay plates were incubated for 5 h at 37 °C in a humidified atmosphere containing 5% CO_2_, followed by the addition of 23 nL test compounds or DMSO vehicle control to assay plates using a Wako Pintool station (Wako Automation, San Diego, CA).

Control placement was established in columns 1 to 4 of each plate. In agonist mode, column 1 contained a final concentration series of positive control, hWNT-3A (0.058–1916.7 ng/mL). Column 2 contained 1916.7 ng/mL hWNT-3A and cell viability positive control (76 µM tetraoctylammonium bromide). For antagonist mode, column 1 contained a concentration series of a known inhibitor, imatinib mesylate (2 nM–76 µM) (Coluccia et al., 2007). Column 2 contained 76 µM imatinib mesylate and 76 µM tetraoctylammonium bromide. In both modes, columns 3 and 4 were DMSO vehicle controls.

Following compound transfer, 1 µL of stimulation solution or buffer was added. In agonist mode, 1 µL of 60 mM LiCl (final concentration 10 mM) was added to all wells except for column 3. For antagonist testing, 1 µL of a stimulation solution, containing 60 mM LiCl, 570 ng/mL hRS, and 450 ng/mL hWNT-3A, was added to all wells except for column 3 (final concentrations in the 6 µL assay volume: 10 mM LiCl, 95 ng/mL hRS, and 75 ng/mL hWNT-3A). For both modes, 1 µL of assay buffer with or without LiCl was added to column 3 as negative control. Plates were incubated overnight (16 h) at 37 °C and 5% CO_2_.

After the 16-h compound treatment, β-lactamase activity was measured by adding 1 µL detection mixture (6 µL CCF4-AM substrate + 60 µL Solution B + 934 µL Solution C). Plates were then incubated for 2.5 h at room temperature, protected from light. Fluorescence was measured on an EnVision plate reader (PerkinElmer, Boston, MA) using excitation at 405 nm and dual emission at 460 and 530 nm. Wnt pathway activation was quantified as the ratio of 460 nm–530 nm emission intensities.

### 2.4 Cell viability assay

To assess compound-induced cytotoxicity, cell viability was assessed using the CellTiter-Glo® One Solution Assay reagent (Promega, Cat #G8462). Following the LEF/TCF-bla assay readout, 4 µL of CellTiter-Glo was added directly to each well. Plates were incubated at room temperature for 30 min to ensure complete cell lysis and stabilization of the luminescent signal. Bioluminescence, generated by luciferase-catalyzed luciferin oxidation and indicative of intracellular ATP content and thus cell viability, was measured on a ViewLux plate reader (PerkinElmer). Compounds reducing bioluminescence by ≥ 30% relative to vehicle controls were flagged as potentially cytotoxic.

### 2.5 qHTS and data analysis

A total of 1,280 compounds from the Library of Pharmacologically Active Compounds (LOPAC; Sigma) and 88 compounds from the Toxicology in the 21st Century (Tox21) 10K compound library were screened. Compound stock plates were prepared with interplate titrations spanning eight concentrations (ranging from approximately 130 μM to 10 mM), with the four left columns for controls. Automated pin tool transfer of 23 nL of compounds to assay plates resulted in a 261-fold dilution, yielding final compound concentrations ranging from 0.5 nM to 38 µM in the 6 µL assay volume.

Primary screening data were processed as previously described (Southall et al., 2009). Raw data for each well were normalized relative to control wells on the same plate. For agonist mode, normalization used DMSO with LiCl wells (basal activity, 0%) and wells treated with a maximal concentration of hWNT-3A (1916.7 ng/mL, defined as 100% activation). For antagonist mode, normalization was performed against DMSO with LiCl wells containing the stimulation solution (LiCl + hRS + hWNT-3A, defined as 0% activity) and wells containing DMSO and LiCl (basal activity, −100%). Normalized data were then corrected for potential plate-pattern artifacts using algorithms based on the signals from compound-free (DMSO) control plates (Wang et al., 2016).

Corrected data points for each compound were fit to the Hill equation to determine the half-maximal activity concentration (EC_50_ or IC_50_) and maximal response (efficacy). Concentration-response curves were classified into categories (1.1, 1.2, 1.3, 1.4, 2.1, 2.2, 2.3, 2.4, 3, or 4 for agonists; analogous classes in negative values for antagonists) based on curve quality, efficacy, and the number of data points above the activity threshold, as previously described (Southall et al., 2009). Curves classified as 1.1, 1.2, 2.1, or 2.2 were considered active, whereas class 4 curves indicated inactive compounds at the concentrations tested. Data from the CellTiter-Glo viability counter screen were analyzed using the same normalization and curve-fitting procedures to identify compounds that caused a significant decrease in bioluminescent signal, indicative of cytotoxicity.

### 2.6 Wnt-luciferase (Wnt-luc) reporter assay for hits confirmation

To validate hit compounds in an independent reporter system, selected compounds were tested using a Wnt-luciferase reporter assay. HEK 293 STF cell line (ATCC, Cat #CRL-3249), which stably express firefly luciferase under the control of LEF/TCF response elements, were seeded in 1,536 well white tissue culture plates at 2000 cells in 4 µL per well in DMEM supplemented with 10% FBS and 1% Pen/Strep. After 24 h incubation at 37 °C and 5% CO_2_ to allow cell attachment, compounds were added at the indicated concentrations (final DMSO concentration ≤0.5%), followed by addition of 1 µL 1,000 ng/mL hWNT-3A (final 200 ng/mL). Plates were incubated for 24 h at 37 °C and 5% CO_2_.

Luciferase activity was measured using ONE-Glo Luciferase Assay reagent (Promega, Cat #E6120) according to the manufacturer’s instructions. Four µL of reagent was added directly to each well, and plates were incubated for 30 min at room temperature. Bioluminescence was measured on a ViewLux plate reader (PerkinElmer). Data were normalized to DMSO + stimulator controls (defined as 0% inhibition) and DMSO without stimulator (defined as 100% inhibition). Concentration-response curves were fitted using a four-parameter variable slope model in GraphPad Prism to determine IC_50_ values, as described in Section 2.8.

### 2.7 Western blot analysis

Western blotting was performed to assess the protein levels of non-phospho (active) β-catenin (Cell Signaling Technology, MA, USA; Cat #8814) and total β-catenin (Cell Signaling Technology, MA, USA; Cat #9562). Cells were seeded in 12-well plates and treated with DMSO alone, DMSO with the stimulator (75 ng/mL hWNT-3A and 95 ng/mL hRS), or 1 µM of each compound (except for cytosine-1-β-D-arabinofuranoside hydrochloride, used at 10 µM) in the presence of the stimulator for 16 h at 37 °C. Each treatment was performed in triplicate. After incubation, the assay medium was removed, and cells were gently washed with PBS.

Cells were lysed with RIPA buffer (Thermo Fisher Scientific, Rockford, USA; Cat #89901) supplemented with a protease and phosphatase inhibitor cocktail (Thermo Fisher Scientific, Rockford, USA; Cat #78440) as described previously (Jovanovic et al., 2024). Cell lysates were immediately collected by scraping into the 1.5 mL microcentrifuge tubes and incubated on ice for 10 min. Samples were clarified by centrifugation at 14,000 × g for 15 min at 4 °C. Protein concentrations were determined using the BCA protein assay kit (Thermo Fisher Scientific, Rockford, USA; Cat #A53225) according to the manufacturer’s instructions. Protein samples were diluted to 500 ng/μL in 4× Bolt LDS Sample Buffer (Thermo Fisher Scientific, CA, USA; Cat #B0007), 10× Sample Reducing Agent (Thermo Fisher Scientific, CA, USA; Cat #B0009), and RIPA buffer, then stored at −30 °C until use.

Equal amounts of protein (5 µg per sample) were loaded onto Bolt Bis-Tris gels for electrophoresis, followed by membrane transfer using the iBlot 2 (Thermo Fisher Scientific). Membranes were blocked and incubated with primary antibodies at 1:1,000 dilution, followed by secondary antibody incubation with HRP-linked anti-rabbit IgG (Cell Signaling, Cat #7074) at 1:5000 dilution. Protein bands were visualized using the G:Box Chemi XX6 imaging system (Syngene, Cambridge, UK) and quantified using ImageJ software (National Institutes of Health, Bethesda, USA). Quantitative results were expressed as mean ± standard error of the mean (SEM). Statistical significance was determined by ordinary one-way ANOVA with Dunnett’s multiple comparison test using GraphPad Prism (GraphPad Prism v10.2.3, CA, USA).

### 2.8 Statistical analysis

Unless otherwise specified, quantitative data are presented as mean ± standard deviation (SD) from at least three independent experiments. Concentration-response curves were fitted using a four-parameter variable slope model in GraphPad Prism.

## 3 Results

### 3.1 Development and optimization of the LEF/TCF-β-lactamase reporter assay

**FIGURE 2:**
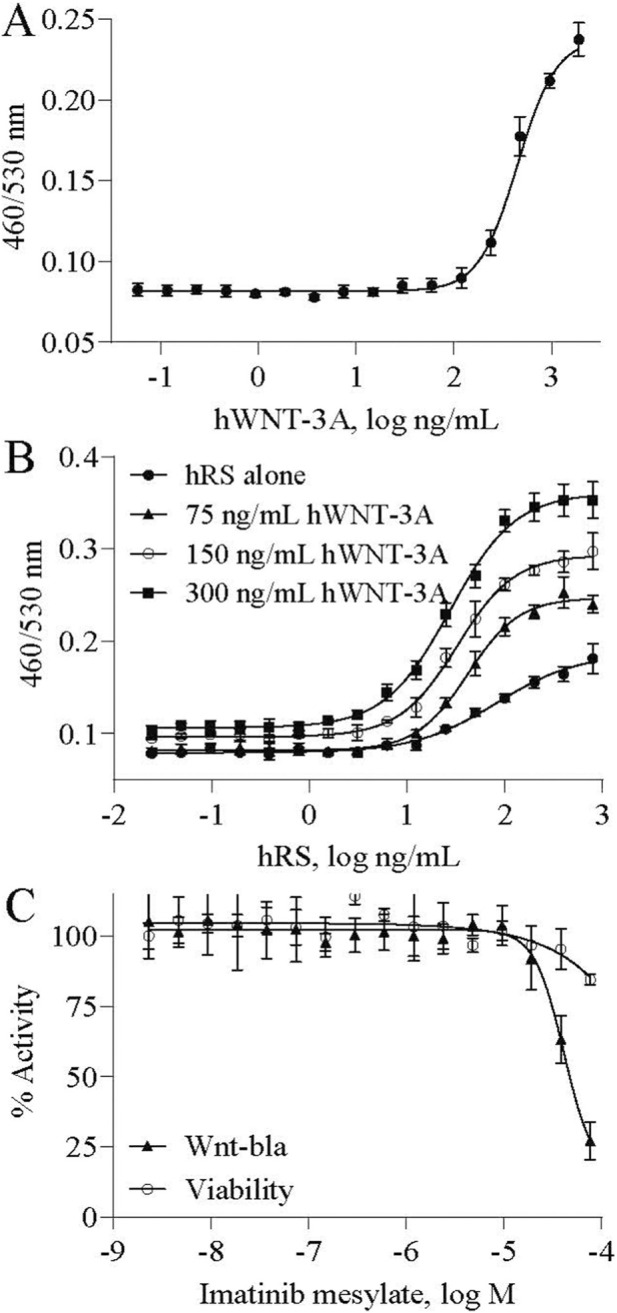
β-lactamase activity concentration-response to compounds in LEF/TCF-bla FreeStyle™ 293F cell in 1536-well format. **(A)** Stimulation concentration response curve of human WNT-3A (hWNT-3A). **(B)** Stimulation concentration response curves of the Wnt potentiator R-Spondin2 (hRS) in combination with different concentrations of hWNT-3A (hRS alone, 75, 150, and 300 ng/mL). **(C)** Curves showing the mild effect of imatinib mesylate on cell viability and its inhibitory effect on stimulator-induced β-lactamase activity, confirming assay responsiveness to known Wnt pathway antagonists.

The LEF/TCF-bla assay was successfully optimized for high-throughput screening in a 1536-well microplate format, enabling the identification of both potential agonists and antagonists of Wnt signaling. In agonist mode, cells treated with hWNT-3A exhibited a robust concentration response relationship, with an EC_50_ value of 449 ng/mL (Figure 2A). This potency aligns with previously reported EC_50_ values (130–250 ng/mL) from similar TCF/LEF reporter assays in HEK293 and related cell systems (Bowin et al., 2019). The optimized assay showed an S/B ratio of 2.8, CV of 7.2%, and a Z-factor of 0.7. A Z-factor between 0.5 and 1.0 indicates an excellent assay with adequate separation between positive and negative controls, while lower values indicate marginal or unsuitable assays for screening (Zhang et al., 1999). These metrics confirm the assay’s robustness for detecting Wnt pathway agonists.

To evaluate the inhibitory potential of test compounds, the assay was configured in antagonist mode by activating the Wnt/β-catenin signaling pathway to generate a good assay window of signal to background. This was achieved by co-treating cells with hWNT-3A and hRS, which amplifies Wnt signaling through stabilization of Frizzled and LRP5/6 receptor complexes *via* LGR4/5 interactions. The optimal concentrations of hWNT-3A and hRS were determined through a concentration-response study using hWNT-3A at hRS alone, 75, 150, and 300 ng/mL. As shown in Figure 2B, the EC_50_ of hRS was significantly reduced in the presence of hWNT-3A, measuring 41.8, 32.6, and 27.5 ng/mL with 75, 150, and 300 ng/mL of hWNT-3A, respectively, substantially lower than the EC_50_ of 82.6 ng/mL observed for hRS alone. Signal to background ratio for the 75, 150, and 300 ng/mL of hWNT-3A conditions were 3.1, 3.1, and 3.5, respectively, which exceeded the 2.3-fold increase observed with hRS alone (Figure 2B). These findings indicate that 75 ng/mL hWNT-3A was sufficient for robust reporter activation when combined with hRS. Assay performance was consistent, with DMSO control CV of 6.7%, 7.2%, 6.9%, and 8.3% for hRS alone, 75, 150, and 300 ng/mL hWNT-3A conditions, respectively. Based on these results, an optimal stimulator combination of 75 ng/mL hWNT-3A and 95 ng/mL hRS (corresponding to the EC_80_ condition) was selected for the antagonist assay. Under these conditions, the known Wnt signaling antagonist imatinib mesylate (Coluccia et al., 2007) was tested, demonstrating an IC_50_ of 41.4 µM in reducing β-lactamase activity, with negligible effects on cell viability (Figure 2C).

### 3.2 LOPAC library screening performance and reproducibility

A total of 1,368 compounds (1,280 from the LOPAC library and 88 from the Tox21 collection) were screened in both agonist and antagonist modes. The antagonist assay utilized 75 ng/mL hWNT-3A and 95 ng/mL hRS as stimulators.

In agonist mode, across 27 plates (Triplicate of eight compound plates and one DMSO control plate), the S/B ratio was 2.8 ± 0.1, CV was 7.9% ± 0.8%, and the Z-factor was 0.6 ± 0.07. In antagonist mode, the S/B ratio was 2.9 ± 0.2, CV was 7.2% ± 1.1%, and the Z-factor was 0.6 ± 0.07. Cell viability measurements further support assay robustness (Table 1).

**TABLE 1:** Assay performance against LOPAC library.

Assay mode	Assay	S/B	Z factor	CV%
Agonist	Wnt-bla	2.8 ± 0.1	0.59 ± 0.07	7.9 ± 0.8
	CellTiter Glo	115 ± 6.6	0.59 ± 0.07	15.6 ± 2.2
Antagonist	Wnt-bla	2.9 ± 0.2	0.58 ± 0.07	7.2 ± 1.1
	CellTiter Glo	135 ± 7.3	0.82 ± 0.05	7.6 ± 1.1

**FIGURE 3:**
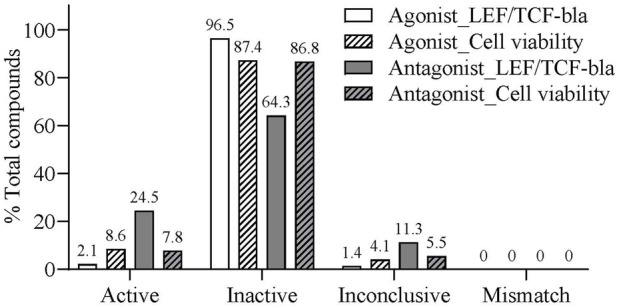
Assay reproducibility of the LEF/TCF-bla reporter assay in a 1536-well format. Bar graphs show the percentage of active match, inactive match, inconclusive match, and mismatch compounds identified across three independent screening replicates (N = 3) in agonist and antagonist modes.

Reproducibility was evaluated by screening the compound library in three independent replicates (Huang, 2022). As shown in Figure 3, the rates of active and inactive matches, and inconclusive compounds in agonist mode were 2.1%, 96.5%, and 1.4%, respectively. In antagonist mode, these rates were 24.5%, 64.3%, and 11.3%. For cell viability, the active and inactive matches, and inconclusive rates were 8.6%, 87.4%, and 4.1% in agonist mode, and 7.8%, 86.8%, and 5.5% in antagonist mode. Importantly, mismatch rates across replicates were 0, thereby demonstrating high reproducibility and internal consistency.

### 3.3 Hit compounds identification and confirmation

From the primary screen, 2 agonists were identified based on criteria of potency <15 μM, efficacy >70%, and presence of accepted concentration-response curves in at least two replicates (classes 1.1, 1.2, 1.3, 2.1, 2.2, or 2.3). In antagonist mode, 19 compounds were identified with potency <1 µM and efficacy <-50%. The most potent inhibitor was phorbol 12-myristate 13-acetate, with an IC_50_ of 0.1 µM.

**FIGURE 4:**
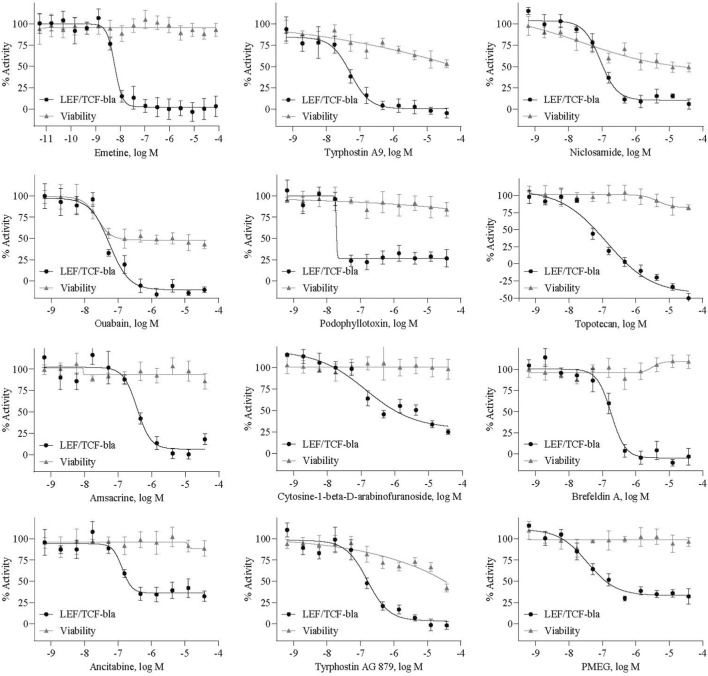
Identification of Wnt/β-catenin signaling pathway inhibitors. Twelve compounds were identified as potential inhibitors of the Wnt/β-catenin signaling pathway based on their mild effects on cell viability and concentration-response analysis using the LEF/TCF-bla reporter assay. All exhibited low IC_50_ values in the micromolar range and inhibitory efficacy greater than 70%. Among these hits, five were previously reported Wnt pathway inhibitors, whereas the remaining seven were newly identified compounds.

Of the 21 active compounds (2 agonists and 19 antagonists), 14 (2 agonists and 12 antagonists) were cherry-picked for confirmation based on compound availability in house. These were re-tested using an independent LEF/TCF-bla assay. All 14 compounds produced their original activity profiles, yielding a 100% confirmation rate (antagonists shown in Figure 4).

Among the confirmed agonists, both BIO (GSK-3 inhibitor IX) and SB 216736 are known Wnt pathway activators. The EC_50_/IC_50_ and efficacy values obtained in both the primary screening and the confirmation assay are listed in Supplementary Table S1.

### 3.4 Orthogonal validation using an independent luciferase reporter system

To exclude the possibility of reporter-specific artifacts and validate hits in an independent system, we tested the 12 potential antagonists using a Wnt-luc reporter assay. Ten of the compounds inhibited the Wnt-luc reporter activity in a concentration-dependent manner. Notably, tyrphostin A9, emetine, niclosamide, brefeldin A, and ouabain exhibited IC_50_ values of 0.48, 0.63, 0.065, 0.21, and 0.79 µM, respectively, in the LEF/TCF-bla assay, and showed corresponding IC_50_ values of 0.81, 0.83, 0.061, 0.25, and 0.48 µM in the Wnt-luc assay. All compound’s IC_50_ values were listed in Supplementary Table S1. These results confirm that the identified compounds are *bona fide* Wnt pathway inhibitors rather than β-lactamase-specific artifacts and demonstrate the robustness and reproducibility of our screening platform across different reporter detection mechanisms.

### 3.5 Validation of potential antagonists via β-catenin protein level analysis

**FIGURE 5:**
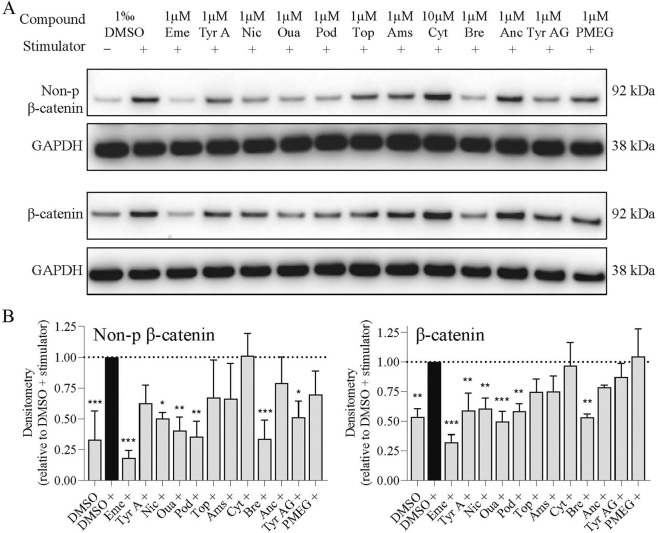
Western blot confirmation of identified inhibitors. **(A)** Representative Western blot analysis showing the effects of each compound on β-catenin protein levels. Treatment with the stimulator (DMSO +) markedly increased both non-phosphorylated (active) and total β-catenin compared with the DMSO control (DMSO -). All known inhibitors attenuated the stimulator-induced increases. **(B)** Quantitative densitometry of non-phosphorylated and total β-catenin from three independent experiments, normalized to the housekeeping protein GAPDH. Data are presented as mean ± SD. Statistical significances relative to the DMSO + control is indicated above bars (*p < 0.05; **p < 0.01; ***p < 0.001). “+” denotes the addition of stimulator; “-” denotes its absence. The stimulator consisted of 75 ng/mL hWNT-3A and 95 ng/mL hRS.

To further validate antagonist activity, β-catenin protein levels were examined by Western blotting, focusing on non-phosphorylated β-catenin (the active form) and total β-catenin. As expected, the DMSO + stimulator group showed significant increases in both forms of β-catenin compared with DMSO-only control (Figure 5A). Ten compounds attenuated stimulator-induced increases, as quantified across three independent replicates (Figure 5B). Notably, emetine, niclosamide, ouabain, podophyllotoxin, and brefeldin A significantly reduced both non-phosphorylated and total β-catenin. Tyrphostin A9 inhibited both non-phosphorylated and total β-catenin, although the effect on the non-phosphorylated β-catenin did not reach statistical significance, while tyrphostin AG 879 significantly suppressed non-phospho β-catenin only. However, cytosine-1-beta-D-arabinofuranoside and PMEG failed to decrease both β-catenin level. These findings demonstrate that the identified antagonists, including emetine, tyrphostin A9, niclosamide, ouabain, topotecan, amsacrine, brefeldin A, and tyrphostin AG 879, primarily reduce non-phosphorylated β-catenin protein levels, providing orthogonal validation of their activity in the reporter assay.

## 4 Discussion

In this study, we successfully optimized a LEF/TCF-bla reporter assay for qHTS in a 1536-well plate format and applied it to screen the LOPAC library. This robust platform led to identification of several Wnt pathway modulators, most notably including topotecan, amsacrine, brefeldin A, and tyrphostin AG 879, as potential antagonists of Wnt/β-catenin signaling. The antagonistic activity of these compounds was consistently confirmed in Wnt-luc assay, demonstrating reproducible potency across two independent reporter systems (LEF/TCF-β-lactamase and LEF/TCF-luciferase) with distinct detection mechanisms. Western blot analysis further validated their activity, showing reduction in both non-phosphorylated (active) and total β-catenin protein levels.

To our knowledge, the identification of topotecan, amsacrine, brefeldin A, and tyrphostin AG 879, as Wnt/β-catenin pathway antagonists has not been previously reported. In addition, our screen also identified several well-characterized Wnt inhibitors, including emetine (Sun et al., 2019; Peng et al., 2023; Wu et al., 2019; Son and Lee, 2021), niclosamide (Osada et al., 2011; Wang et al., 2019; Chen et al., 2009; Monin et al., 2016), ouabain (Tejeda-Muñoz et al., 2024), podophyllotoxin (Kalita et al., 2019), and tyrphostin A9 (Miller et al., 2012), thereby validating the reliability and pathway specificity of our screening platform despite the use of a reporter-based assay. The identification of these known inhibitors provides retrospective confirmation that the assay detects *bona fide* Wnt pathway modulators and supports the classification of topotecan, amsacrine, brefeldin A, and tyrphostin AG 879 as additional chemical probes for investigating Wnt/β-catenin pathway.

The mechanism by which brefeldin A suppresses Wnt signaling warrants further elucidation. Brefeldin A is a well-established inhibitor of vesicular trafficking, acting primarily through its inhibition of ADP-ribosylation factor (ARF)-dependent guanine nucleotide exchange factors (GEFs), which in turn disrupt endoplasmic reticulum (ER)-to-Golgi transport (Helms and Rothman, 1992; Klausner et al., 1992; Chardin et al., 1999). It is plausible that brefeldin A interferes with trafficking-dependent processes essential for Wnt pathway activation (Port et al., 2008; Werner et al., 2023; Napolitano et al., 2023; Solis et al., 2013). Wnt ligands, being secreted glycoproteins (Port et al., 2008), require synthesis in the ER, modification by Porcupine (PORCN) in the ER, and subsequent transport through the Golgi apparatus for proper processing and secretion (MacDonald et al., 2009; Port et al., 2008; Takada et al., 2017). By blocking ER-to-Golgi transport, brefeldin A could prevent Wnt ligand secretion, thereby inhibiting downstream signaling (Werner et al., 2023; Napolitano et al., 2023). Similarly, proper localization of Wnt receptors such as the Frizzled family depends on intact vesicular trafficking; disruption by brefeldin A could impair receptor availability at the cell surface and hinder signal transduction (Werner et al., 2023; Solis et al., 2013). Interestingly, brefeldin A inhibits Wnt signaling with an IC_50_ of approximately 0.2 µM, which is 85- to 170-fold lower than concentrations required to disrupt general vesicular trafficking (commonly 5–10 μg/mL or 18–36 µM) (Lippincott-schwartz et al., 1989; Fujiwara et al., 1988; Low et al., 1991; Sciaky et al., 1997). Consistent with prior findings, protein secretion and Golgi structure in MDCK cells remain largely unaffected at 0.5 μg/mL (1.8 µM), but are markedly impaired at higher concentrations of 10–30 μg/mL (36–107 µM) (Low et al., 1991). This suggests that specific trafficking events required for Wnt activation, such as ligand secretion, receptor transport, or endosomal signaling, may be disproportionately sensitive to partial ARF-GEF inhibition compared with global vesicular transport.

Topotecan and amsacrine, clinically used topoisomerase inhibitors, primarily target on topoisomerase I and II, respectively (Kollmannsberger et al., 1999; Ketron et al., 2012). In our system, both compounds attenuated hWNT-3A-induced both LEF/TCF-controlled reporters’ activity and β-catenin accumulation. Similarly, tyrphostin AG 879, a tyrphostin-class tyrosine kinase inhibitor, functioned as an inhibitor of canonical Wnt/β-catenin signaling in our assays. The decrease in active β-catenin indicates that these compounds interfere with ligand-induced β-catenin stabilization and/or transcriptional competency, rather than simply suppressing reporter gene expressions nonspecifically. Topoisomerase inhibition could alter transcriptional elongation dynamics, affect chromatin accessibility at Wnt-responsive loci, or indirectly influence upstream signaling events required for β-catenin stabilization. The concordant suppression of active β-catenin accumulation and two independent Wnt reporter systems supports a pathway-level effect, though further mechanistic dissection will be required.

Several additional hit compounds, e.g., cytosine-1-beta-D-arabinofuranoside and PMEG suppressed reporter activity without decreasing β-catenin protein levels. This dissociation suggests either alternative mechanisms of Wnt pathway inhibition or potential reporter artifacts. These compounds may act through interference with β-catenin nuclear import, disruption of β-catenin-LEF/TCF complex formation, or direct effects on the reporter construct. The ability to distinguish such compounds from those that reduce endogenous β-catenin levels demonstrates the value of orthogonal validation using Western blot analysis.

Despite the successful identification of Wnt modulators, several limitations should be noted that affect the interpretation and generalizability of our findings. First, the HEK293-derived reporter cell line, while well-suited for high-throughput screening due to its robust reporter expression and established pharmacological response characteristics, is not a Wnt-dependent system. To address potential reporter-specific artifacts, we validated all hit compounds using an independent Wnt-luciferase reporter system, which confirmed their activity with consistent IC_50_ values (Supplementary Table S1). This cross-validation using two distinct reporters (β-lactamase FRET-based vs. luciferase bioluminescence-based) excludes artifacts specific to either detection system. Western blot analysis confirmed a decrease in endogenous β-catenin with topotecan, amsacrine, brefeldin A, and tyrphostin AG 879, and other key hit compounds treatment, providing orthogonal validation beyond the reporter systems. In the future study, it is worth to assess their impact on endogenous canonical Wnt transcriptional output (such as *AXIN2*, *LEF1*, or *MYC* gene expression) (Jho et al., 2002), or their activity in more complex Wnt-dependent cellular models (e.g., organoids) or *in vivo* systems (Cleve et al., 2012). Second, the use of imatinib mesylate as a positive control in the antagonist assay, while supported by published evidence of its effects on β-catenin stabilization (Coluccia et al., 2007), may not constitute an optimal pathway-specific control. Future iterations of this assay would benefit from the inclusion of well-characterized, mechanism-based Wnt inhibitors such as PORCN inhibitors (e.g., LGK974, IWP-2) or tankyrase inhibitors (e.g., XAV939) to enhance confidence in pathway-specific hit identification. Finally, because several identified compounds, including topotecan, amsacrine, brefeldin A, and tyrphostin AG 879, have broad cellular activities, their Wnt-inhibitory effects may reflect indirect or pleiotropic mechanisms. Further studies should clarify the molecular basis of their effects, including direct evaluation of Wnt ligand secretion, receptor localization, destruction complex dynamics, endogenous target gene expression, and specificity within the Wnt pathway compared to general cellular processes. Such mechanistic studies, ideally conducted in physiologically relevant Wnt-dependent systems, will be essential to determine whether topotecan, amsacrine, brefeldin A, and tyrphostin AG 879 represent a pathway-specific effects or primarily reflect their broader cellular effects.

In summary, our findings provide two key contributions: (Liu et al., 2022): the optimized 1536-well LEF/TCF-β-lactamase assay demonstrates robust performance with standard parameters (Z-factor > 0.5, low CV) and is suitable for expanded compound screening; the identification of multiple known Wnt inhibitors and cross-validation demonstrates the platform’s ability to detect pathway modulators despite the inherent limitations of reporter-based assays; and (Klaus and Birchmeier, 2008) topotecan, amsacrine, brefeldin A, and tyrphostin AG 879 are identified as potential antagonists of Wnt/β-catenin signaling that warrants further mechanistic investigation. While their broad cellular effects may limit their therapeutic potential, these compounds represent valuable chemical probe for dissecting Wnt pathway regulation when supported by appropriate controls and orthogonal validation assays. These findings underscore the utility of quantitative high-throughput chemical biology approaches in expanding the repertoire of small molecules available to interrogate complex signaling pathways such as Wnt/β-catenin, paving the way for deeper insights into their biological and pathological roles.

## Data Availability

Original data generated and analyzed during this study are included in this published article, the supplementary material listed in “References,” or are available from the corresponding authors on reasonable request.
